# Post-electric Burn Injury to the Calvarium: A Case Report of a High-Tension Wire Burn With Bone Penetration

**DOI:** 10.7759/cureus.69430

**Published:** 2024-09-14

**Authors:** Sankalp Goel, Alok Sharma

**Affiliations:** 1 Plastic Surgery, Dr. D. Y. Patil Medical College, Hospital and Research Centre, Dr. D. Y. Patil Vidyapeeth (Deemed to be University), Pune, IND

**Keywords:** burn injury, burns scar, calvarium, electric injury, lightening injury

## Abstract

Electrical injuries can be horrifying, leading to physical trauma to the patient, along with emotional and financial trauma to the patient and their relatives. Electrical injuries to the calvarium are especially devastating, particularly when they involve high-voltage electricity. Electric injuries can be associated with multiple types of trauma, including burns, scars (Lichtenberg figures), and fractures resulting from falls or muscular spasms. We present a unique case report of post-electric burns (caused by a high-tension wire) over the calvarium, resulting in a lightning-shaped burn partially penetrating the bone. The effect of electrical injury on bone is less well understood, and evidence of such burn injuries should be made aware.

## Introduction

In cases of high-tension electrical burns (exceeding 1000 V), the extent of injury is largely influenced by the resistance encountered at the point of contact with the electrical source. The transformation of electric energy into heat energy occurs as the current traverses through the body, determining the severity of tissue damage [1]. Extensive electrical burns to the scalp, affecting the entire thickness of the skull, are rare events [1,2]. Severe electrical burns on the scalp manifest when a person's head makes contact with a high-tension wire while another part of the body serves as a grounding point. The electrical current penetrates the body at the point of contact, following paths of least resistance, and exits at the grounding site. Bones have the most resistance, whereas blood vessels and nerves have the least. The severity of the burn depends on the heat's intensity and duration. Initial observations may underestimate the actual size of the burns, as thrombosis of nearby vessels leads to ischemia and sloughing, which is not immediately apparent [3]. A scalp electrical burn takes on a crescent or saucer shape, with the deepest area at the center and a gradual decrease in depth towards the periphery [1].

## Case presentation

**Figure 1:**
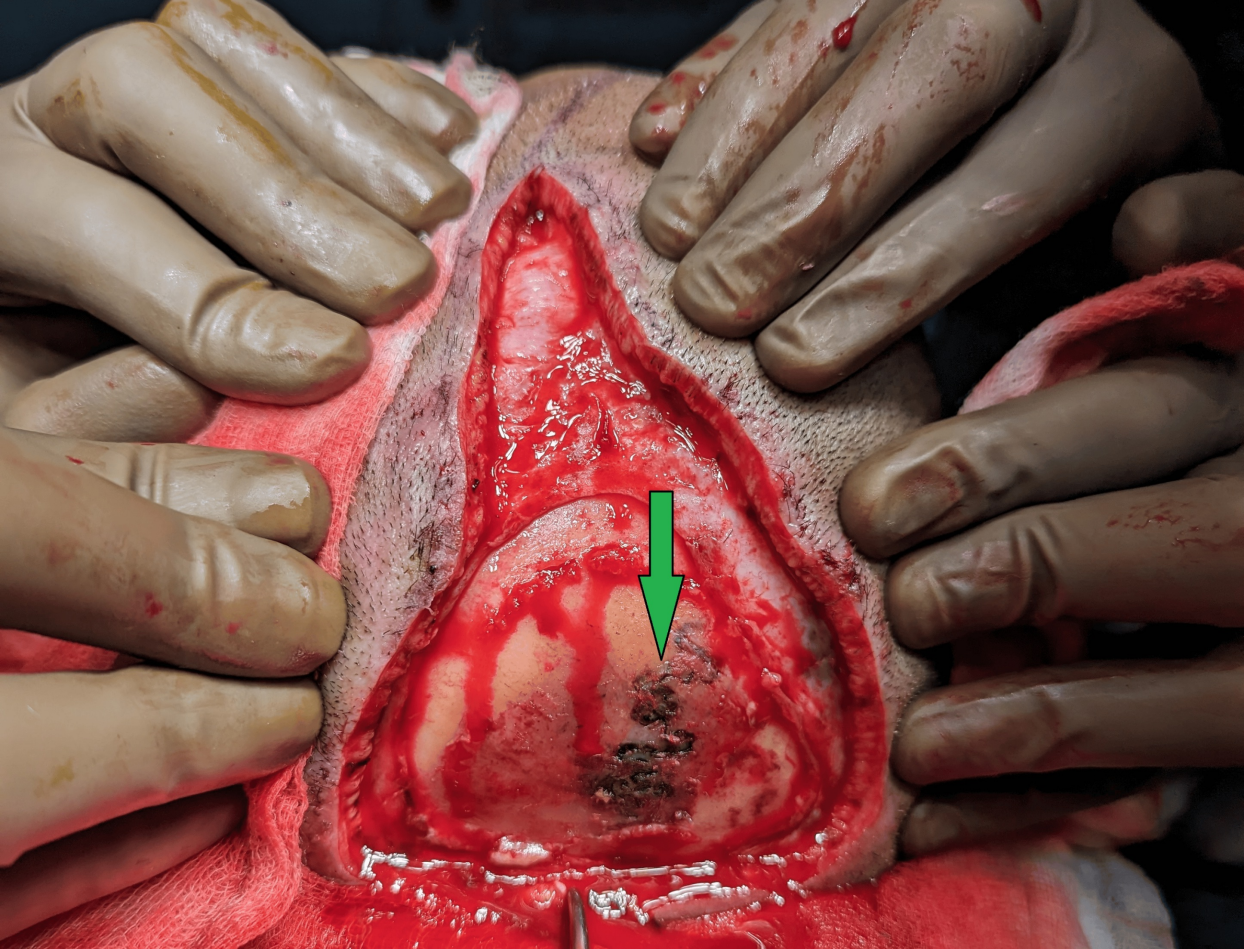
Lightening shaped electrical burn to the scalp surrounded by desiccated bone (green colored arrow)

**Figure 2:**
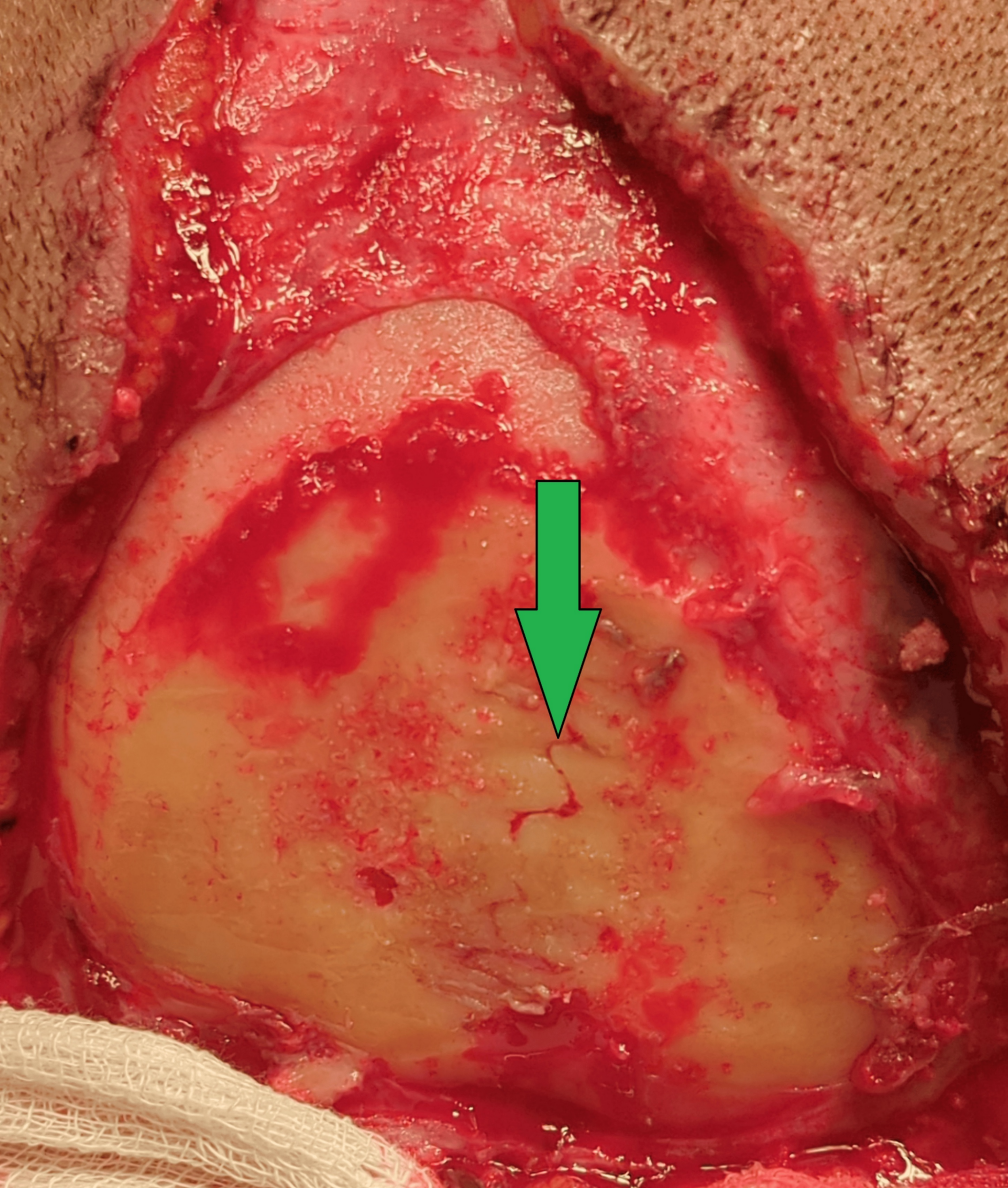
Post-debridement, revealing partial penetration of the burn to the calvarium (green colored arrow)

**Figure 3:**
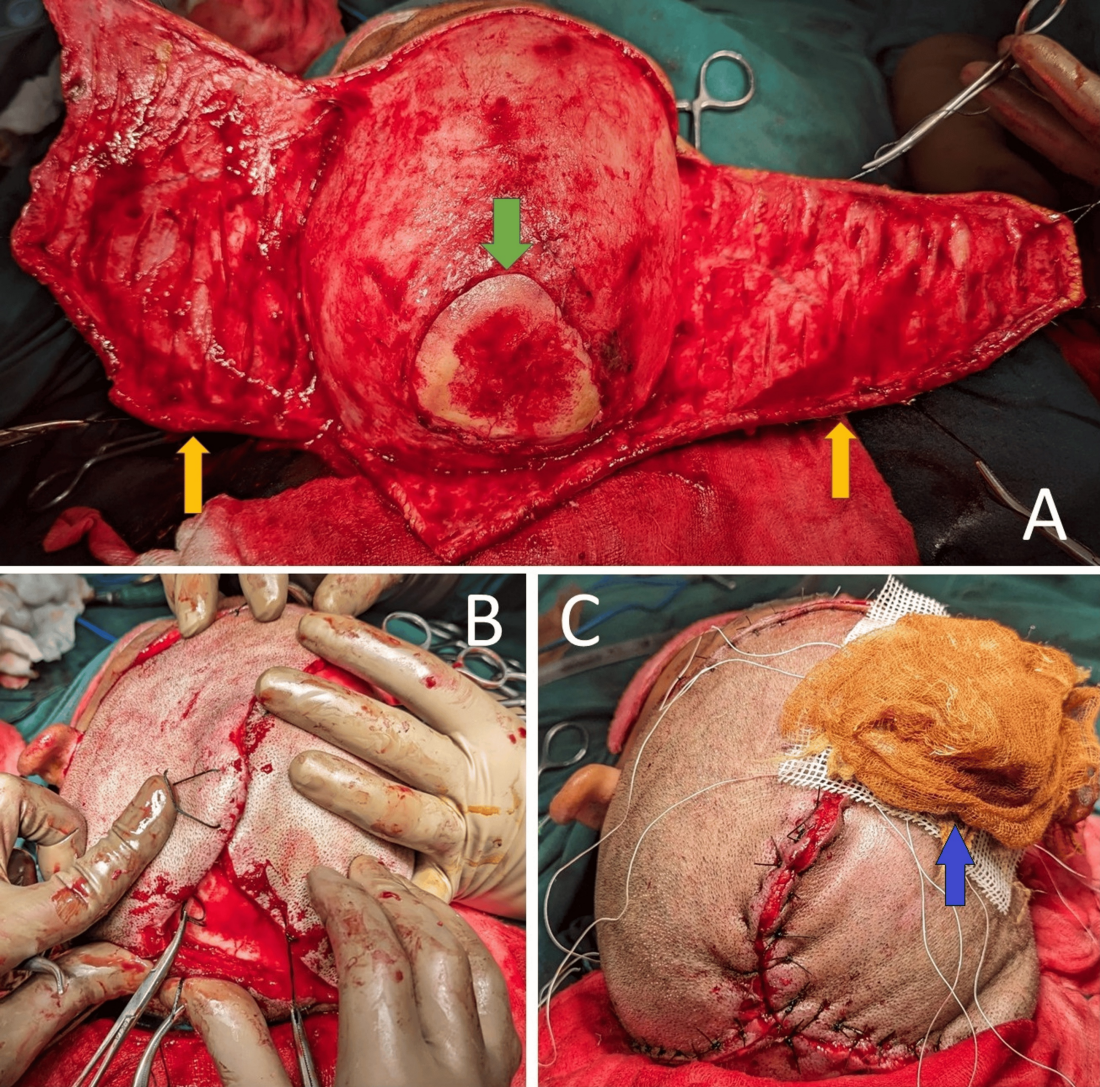
Operative step while closing the defect with an Orticochea flap A) Elevated Orticochea flaps with the defect; B) Flaps covering the defect; C) Final closure of the defect with STSG and tie-over dressing over resultant raw area Green colored arrow: Defect; Yellow colored arrows: Orticochea flaps; Blue colored arrow: STSG with tie-over dressing STSG: Split-thickness skin graft

A 40-year-old female was working on her farm under multiple high-tension electrical wires that traversed her fields. One of the wires snapped and dropped onto her scalp. She was freed from the contact by her daughter, who also suffered injuries to the scalp. She did not experience any loss of consciousness, seizure, vomiting, or ENT bleeding. She was rushed to the hospital, where she was managed conservatively and dressed. She presented to us one month after the trauma, with a triangular wound over the scalp measuring 20 cm in length and 15 cm in breadth, with loss of pericranium measuring 10 cm by 15 cm, and an eschar covering the wound. There was a well-granulating raw area surrounding the eschar. Her CT scan of the head showed soft tissue loss over the calvarium, with no abnormalities detected in either the brain or bone. On culture, she tested positive for Methicillin-resistant *Staphylococcus aureus*, which was sensitive to linezolid, sulfamethoxazole, and trimethoprim. After an adequate duration of antibiotic coverage and regular dressings, the patient was taken for flap cover with an Orticochea flap. On debridement of the wound, a lightning-shaped burn was seen on the right parietal region (Figure 1). The unviable bone was burred to unveil that the depth of the scar was near total (Figure 2). Flaps were raised, and the whole defect was covered, with the resultant raw area (anteriorly) covered with a split-thickness skin graft (STSG) harvested from the right thigh, and a tie-over dressing was applied (Figure 3). The procedure was uneventful, and the patient was discharged with sutures in situ on postoperative day 10 (after graft inspection) and was asked to keep in constant follow-up. Post-operative follow-ups were uneventful, and the wound healed completely with no complications.

## Discussion

Electrical injuries have been documented involving the skin, fascia, muscle, and visceral organs, but there is limited literature on electrical injury to bone. On the skin, there are reports of fern-shaped Lichtenberg figures, as described by Nagesh et al. [4] and Raniero et al. [5]. These figures are considered to be pathognomonic for lightning exposure to the skin. Mostly unknown, they are thought to result from leakage through damaged vessels while the skin is moist. They are temporary lesions that last up to 48 hours [6]. Celiköz et al. reported a full-thickness burn to the scalp, involving all layers of the skin and the entirety of the bone, but not involving any intracranial structures in the case of a lightning strike to the scalp. The bone was debrided down to the dura mater, which was intact, and covered with a latissimus dorsi flap [7]. 

In our case, the injury to the bone was not apparent on CT scans. On exposure, the bone had a lightning bolt-shaped burn, not unlike the lightning bolt scar of Harry Potter, penetrating partially through the calvarium. This is especially dangerous, as this area overlaps the superior sagittal sinus, which, if injured, could be fatal. Electrical injuries to bone are less understood.

To help better understand the impact of electrical injuries on the bone, especially those from high-voltage injuries such as lightning strikes, Bacci et al. conducted an experiment simulating damage to the bone when exposed to high-voltage electricity. The simulation showed different patterns of microfractures that emerged with lightning exposure to the bone, ranging from interstitial microfractures to complete shattering of the bone [8].

Brinn and Moseley have reported that, due to the high resistance of bone to electrical energy, the bone heats to over several thousand degrees Celsius. The heat can lead to necrosis of the bone and the surrounding tissues [9]. This explains the saucerization of electrical injuries, where the burns are deeper in the center and, as heat dissipates peripherally, the burns reduce.

**Figure 4:**
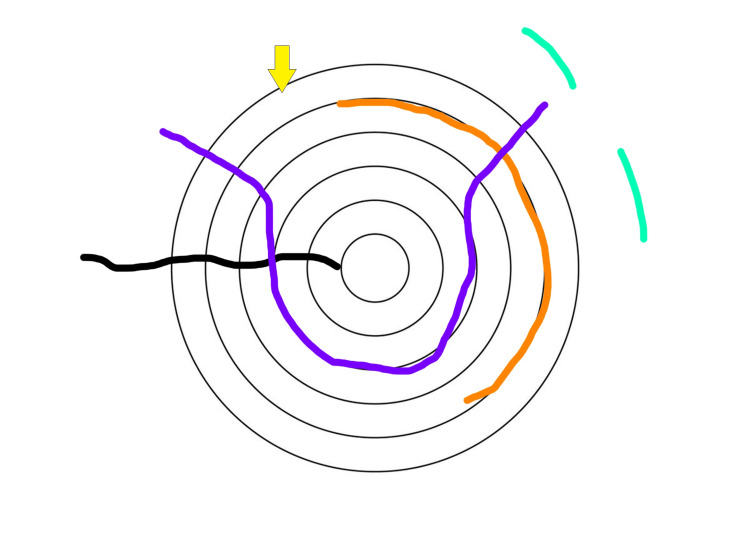
A representation of possible microfractures due to electrical injury to the bone (yellow arrow) Bone (yellow colored arrow); Circumferential (orange colored line); Circumferential irregular (purple colored line); Radiating (black coloured line); Interstitial (green coloured line)

Since bone has such a high resistance, the electrical energy finds a path of least resistance, which has resulted in a lightning-shaped burn over the calvarium. The burn has traveled along the sagittal suture, which is probably where the resistance of the bone is the least, leading to a burn in a similar shape. There have been patterns of microfractures in the bone, as shown in Figure 4. Macrofractures have been noted in individuals post-electrical injury due to either impact after a fall or severe muscle spasm leading to a long bone fracture. The burn injury we see here is a direct result of the electrical energy, which has partially penetrated the calvarium. This is of particular concern, as the penetrating burn injury was along the sagittal suture, which overlies the superior sagittal sinus. If the superior sagittal sinus is injured, it can lead to severe morbidity or, worse, mortality.

## Conclusions

Electrical injuries to the scalp are potentially under-evaluated, which may also be due to CT scan reports. Although wounds may appear superficial, the managing physician should consider the thickness of the scalp and cranial vault, as well as the possibility of their involvement. High-voltage electricity has the potential to penetrate deeper structures, possibly involving the superior sagittal sinus or the dura mater. All such cases should be evaluated thoroughly, considering the possibility of injury depth.
